# Optimization of the Synthesis of Low Viscosity and High Shear Sulfonated Guar Gum for Enhancing Its Performance in Drilling Fluids

**DOI:** 10.3390/gels11120939

**Published:** 2025-11-22

**Authors:** Yifei Zhao, Yansong Pan, Le Xue, Yongfei Li, Weichao Du, Gang Chen

**Affiliations:** 1 Engineering Research Center of Oil and Gas Field Chemistry, Xi’an Shiyou University, Xi’an 710065, China; 190606@xsyu.edu.cn (Y.Z.); 22212071107@stumail.xsyu.edu.cn (Y.P.); yfli@xsyu.edu.cn (Y.L.); duweichao@xsyu.edu.cn (W.D.); 2 Shaanxi Province Key Laboratory of Environmental Pollution Control and Reservoir Protection Technology of Oilfields, Xi’an Shiyou University, Xi’an 710065, China; 3 School of Petroleum Engineering, China University of Petroleum (Huadong), Qingdao 266580, China; bz25020011@s.upc.edu.cn

**Keywords:** sulfonated guar gum (SGG), low viscosity, high shear, drilling fluids

## Abstract

Guar gum (GG) is a classic polysaccharide gel former in drilling fluids, but its native network is hindered by high water-insoluble residue, modest yield-point (YP) build-up and poor tolerance to temperature ≥ 120 °C and salinity ≥ 12 wt% NaCl. Here we transformed GG into a sulfonated guar gum (SGG) hydrogel via alkaline etherification with sodium 3-chloro-2-hydroxy-propane sulfonate. FTIR, EA and TGA corroborate the grafting of –SO_3_^−^ groups (DS = 0.18), while rheometry shows that a 0.3 wt% SGG aqueous gel exhibits 34% higher YP/PV ratio and stronger shear-thinning than native GG, indicating a denser yet still reversible three-dimensional network. In 4 wt% Ca-bentonite mud the SGG gel film reduces API fluid loss by 12% and maintains YP/PV = 0.33 after hot-rolling at 120 °C, a retention 4.7-fold that of GG; likewise, in 12 wt% NaCl brine the gel still affords YP/PV = 0.44, evidencing electrostatically reinforced hydration layers that resist ionic compression. Linear-swell tests reveal shale inhibition improved by 14%. The introduced –SO_3_^−^ functions strengthen inter-chain repulsion and water binding, yielding a thermally robust, salt-tolerant polysaccharide gel network. As a green, high-performance gel additive, SGG offers a promising route for next-generation water-based drilling fluids subjected to high temperature and high salinity.

## 1. Introduction

Guar gum, a natural and environmentally friendly polymer, has been extensively investigated for oil exploitation applications owing to its biodegradability, availability, and inherent viscosifying properties [1,2,3,4,5]. The viscosifying effects of polysaccharides like corn fiber gum underscore the importance of intermolecular interactions in modulating solution rheology, principles that are directly relevant to guar gum systems [6]. Native guar gum and its derivatives, such as cationic and fluorinated guar gels, exhibit desirable pseudoplastic and shear-thinning behavior, which is effectively described by the Ostwald–Dewael model and is crucial for performance under high-shear drilling conditions [7]. However, the practical deployment of guar gum in challenging environments necessitates enhancements to its thermal and rheological stability. This has been addressed through various modification strategies. For instance, chemical grafting—as demonstrated with xanthan gum copolymers (XG-AA/AM/AMPS and XG-AAA)—imparts high-temperature resistance up to 180 °C, improving shear-thinning behavior and low-shear-rate viscosity, which are vital for cuttings transport and wellbore stability [8,9]. Similarly, cross-linking guar gum with nanosilica particles has been shown to enhance the rheological and breaking properties of fracturing fluids, indicating a viable route to robust fluid design [10]. Research on multicomponent thickening systems further confirms that guar gum-based fracturing fluids can be specifically optimized for rheological stability, a critical requirement for low-permeability reservoirs where maintaining performance under high shear is essential [11].

The rheological performance of guar-based systems is further augmented by synergistic interactions with advanced additives. Incorporating nanoparticles, such as poly (sodium P-styrene sulfonate)-modified Fe_3_O_4_ or graphene oxide, significantly improves rheological stability, filtration control, and shear-thinning characteristics in water-based drilling fluids [12,13]. The influence of thermal conditions on these systems is critical, as evidenced by studies on the variation in rheological properties with temperature in fluids containing natural additives like rice husk [14]. Other additives, including carboxylated cellulose nanocrystals (CNCs) and attapulgite clay, function as effective and eco-friendly rheological modifiers, enhancing fluid performance under high-temperature and high-salinity conditions [15,16]. The gel stability of suspensions like calcium bentonite in brine is also markedly improved by water-soluble polymers or modified vegetable gums, enabling reliable application in saline drilling environments [17]. Beyond particle and polymer additives, alternative fluid systems have been explored, including surfactant-based viscoelastic fluids as non-polymeric alternatives to guar gels [18] and stable foam systems formulated with surfactants like ammonium alcohol ether sulfate for underbalanced drilling [19]. Moreover, the incorporation of hydroxyl-group-bearing thermodynamic hydrate inhibitors can enhance the rheological performance of guar-based fracturing fluids under high-pressure conditions [20].

A comprehensive understanding of the rheological behavior of these complex fluids is essential for optimizing their performance. Studies on the nonlinear viscoelastic properties of bentonite- and sepiolite-based fluids under large amplitude oscillatory shear (LAOS) reveal critical gel-like responses that inform fluid design under dynamic downhole conditions [21]. The Herschel–Bulkley model has been successfully applied to characterize the rheological behavior of additive-enhanced fluids, including those containing attapulgite and polymer-modified nanoparticles [12,16]. Furthermore, the development of high-temperature gelling agents, such as amphiphilic multiblock polymers for oil-based fluids, novel amide compounds for ultra-low-temperature applications, and Mixed metal hydroxide gels as effective high-temperature filtration loss agents, continues to extend the operational limits of drilling fluid gels [9,22,23,24]. Moreover, lignosulfonates are employed in drilling fluid formulations as multifunctional additives, such as fluid loss reducers, inhibitors, and stabilizers [25,26]. Collectively, these advances underscore the significance of tailoring both rheological models and chemical compositions to design next generation drilling fluids with enhanced performance and broader operational windows.

This work demonstrates fundamental distinctions from and innovations beyond prior research in three key aspects. First, it marks a strategic shift in research focus from “viscosity enhancement” to “ shear improvement “. While numerous studies on guar gum and its sulfonated derivatives have concentrated on their use as thickeners in fracturing fluids to increase apparent viscosity, this study is the first to position sulfonated guar gum specifically as a rheology modifier for drilling fluids [27,28,29,30,31]. Our work systematically optimizes the yield point and the yield point-to-plastic viscosity ratio, aiming to enhance cuttings transport without significantly increasing viscosity, thereby establishing a tailored “low-viscosity, high-shear” rheological profile—an approach previously unreported in the literature. Second, it addresses a critical transition in the research medium—from aqueous solutions to complex slurry systems. Whereas fracturing fluid studies are typically conducted in aqueous solutions, drilling fluid performance must be evaluated in slurries, where the complex composition leads to fundamentally different polymer behavior. Consequently, existing data from fracturing fluid studies are not directly applicable and may even be contradictory, a gap that this study systematically addresses. Third, it achieves a dual breakthrough in performance and cost-effectiveness. Conventional rheology modifiers, such as xanthan gum, carry approximately twice the cost of guar gum and often cause undesirable viscosity increases. In contrast, sulfonated guar gum successfully decouples shear enhancement from viscosity buildup while offering significant cost reduction, thereby delivering superior functionality alongside enhanced economic viability.

To address the current research gap, this study focuses on the synthesis and systematic evaluation of SGG as a targeted rheology modifier. The sulfonation modification is designed to introduce sulfonate groups onto the guar backbone, which are anticipated to significantly bolster its hydration capability and electrostatic repulsion, thereby fostering a more stable gel network in demanding environments. The present work meticulously optimizes the sulfonation reaction parameters and provides a multi-faceted performance assessment, demonstrating that SGG effectively delivers a favorable “low-viscosity, high-shear” profile and enhanced retention of key rheological properties after exposure to high temperature and high salinity. Our findings confirm that sulfonation is a highly effective strategy to engineer guar gum into a high-performance gel additive, contributing to the development of more efficient and reliable drilling operations in challenging reservoirs.

## 2. Results and Discussion

**Figure 1:**
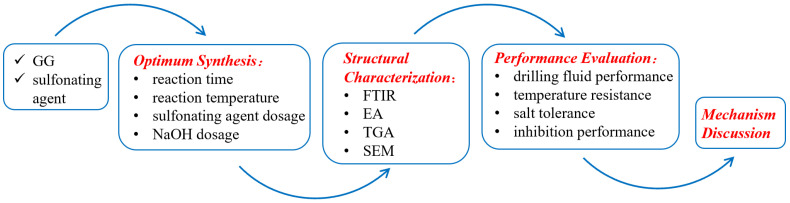
Experimental workflow for the synthesis and evaluation of SGG.

To clearly present the logical framework of this research, the overall experimental workflow is schematically illustrated in Figure 1. It encompasses three major phases: the optimized synthesis and structural characterization of SGG, followed by a systematic evaluation of its performance in both gel solution and drilling fluid applications.

### 2.1. FTIR Analysis

**Figure 2:**
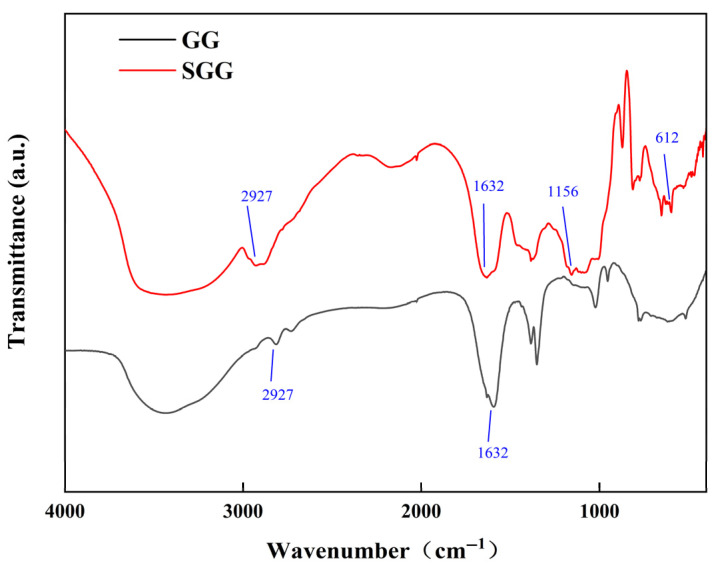
FTIR spectra of GG and SGG.

To confirm the structural and functional group changes after the modification of GG and to verify the impact of sulfonation, FTIR analysis was performed on both GG and SGG. The FTIR spectra (Figure 2) revealed significant changes upon sulfonation. A broad and intense absorption band at 3438 cm^−1^ is attributed to the O–H stretching vibration influenced by intermolecular hydrogen bonding, which is characteristic of natural polysaccharides like GG. The sharp band at 2927 cm^−1^ corresponds to the symmetric and asymmetric C–H stretching vibrations of methylene groups in the sugar units, while the peak at 1632 cm^−1^ arises from the ring stretching vibration of β-(1→4) glycosidic linkages in the galactomannan backbone.

Notably, new absorption peaks emerged at 1156 cm^−1^ and 612 cm^−1^ in the spectrum of SGG. By comparison with standard IR databases of sulfonated compounds, the peak at 1156 cm^−1^ can be assigned to the asymmetric stretching vibration of S=O bonds in the sulfonate groups, and the absorptions near 612 cm^−1^ and 530 cm^−1^ are associated with internal vibrational modes of the sulfonate groups. The appearance of these characteristic peaks provides clear evidence for the successful grafting of sulfonate groups onto the GG backbone [32]. Combined with changes in the relative intensity and position of characteristic absorption bands, these results confirm the successful sulfonation of the GG molecular chains.

### 2.2. EA

The elemental analysis results (Table 1) clearly indicate the successful introduction of sulfonate groups into the GG structure. The S content increased significantly from 0.621% in GG to 0.792% in SGG, representing a 27.5% enhancement. This substantial rise in S content provides direct evidence for the incorporation of sulfonate groups during the modification process. Concurrently, slight decreases were observed in the C (from 39.800% to 38.500%) and H (from 5.998% to 5.345%) contents, which can be attributed to the dilution effect caused by the introduction of sulfonate groups. The N content remained relatively stable, indicating that the sulfonation reaction specifically targeted the hydroxyl groups of the GG backbone without affecting its fundamental structure.

### 2.3. TGA

**Figure 3:**
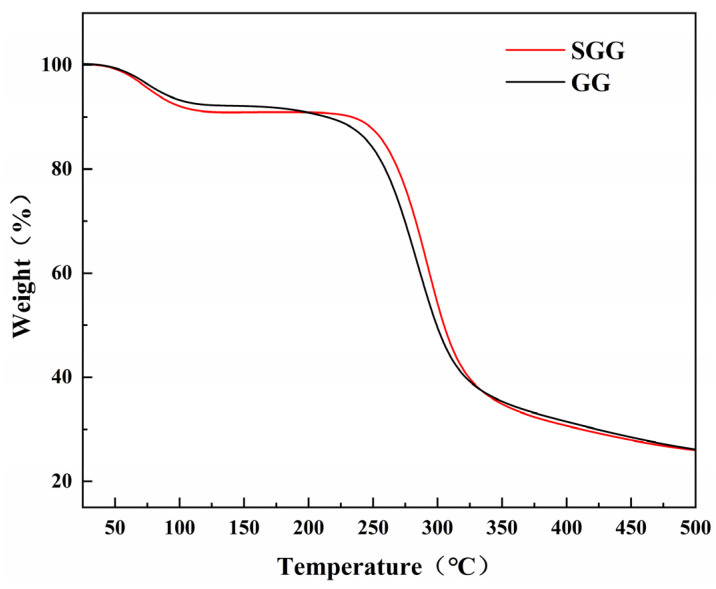
TGA curves of GG and SGG.

Thermogravimetric analysis (TGA) was carried out to evaluate the thermal stability and decomposition behavior of both GG and SGG. Prior to testing, the samples were dried at 60 °C for 6 h. As shown in the TGA curves (Figure 3), the thermal decomposition of both gums occurred in three distinct stages. In the first stage (T ≤ 140 °C), the weight loss was attributed to moisture evaporation. The SGG exhibited a slightly higher weight loss near 100 °C, likely due to its increased hydrophilicity and moisture absorption capacity resulting from the introduced sulfonate groups. The second stage (230–350 °C) involved a significant mass loss of over 50%, corresponding to the decomposition of the polysaccharide backbone through dehydration and scission of glycosidic linkages, leading to the formation of volatile products. Notably, the thermal decomposition peak of the sulfonated derivative in this region shifted to a higher temperature compared to that of GG, indicating enhanced thermal stability of the gel network due to the chemical modification. In the final stage (T > 350 °C), the samples underwent carbonization, during which the residual char continued to degrade slowly.

### 2.4. SEM Analysis

**Figure 4:**
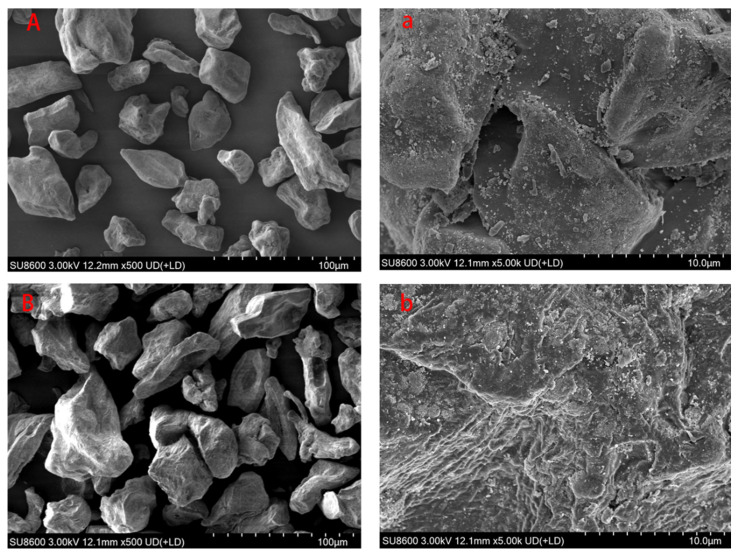
SEM images of GG (**A**,**a**) and SGG (**B**,**b**).

Scanning electron microscopy (SEM) revealed distinct morphological differences between the GG and SGG (Figure 4). The GG (Figure 4A) exhibited discrete, irregularly elongated particles with smooth surfaces and no significant structural defects (Figure 4a), consistent with previously reported morphological characteristics of GG. In contrast, the SGG (Figure 4B) showed altered particle morphology, developing an uneven and corroded-like surface texture (Figure 4b). This surface roughening is likely attributed to the alteration of the crystalline structure of GG under the alkaline conditions employed during sulfonation. This surface roughening and morphological alteration, indicative of changes in the crystalline structure, are consistent with successful chemical modification and likely contribute to the enhanced rheological properties observed.

### 2.5. Optimization Synthesis of SGG

Having confirmed the successful synthesis and enhanced properties of SGG through the foregoing structural characterization, we then focused on optimizing the reaction conditions to achieve the highest performance.

The sulfonation modification of GG is influenced by several factors. To systematically investigate these, a one-factor-at-a-time experimental approach was employed to evaluate the effects of reaction time, reaction temperature, sulfonating agent dosage, and NaOH dosage on the modification efficiency. It is noted that while the one-factor-at-a-time approach effectively identified the individual optimal ranges for each parameter, it does not account for potential interactions between variables. Future studies will employ multivariate optimization techniques to explore these interactions and further refine the synthesis process.

The performance was assessed based on the YP/PV and AV of a 0.3 wt% aqueous solution prepared from the final SGG powder. The YP/PV is a critical indicator for evaluating rheology modifiers, as a favorable (or higher, within an optimal range) value enhances hole-cleaning efficiency and reduces the risk of barite sag while maintaining manageable equivalent circulation densities. Meanwhile, the AV of the gel solution correlates with the water-insoluble content of the GG, serving as an important measure of its quality.

#### 2.5.1. Effect of Reaction Time on the Performance of SGG

**Figure 5:**
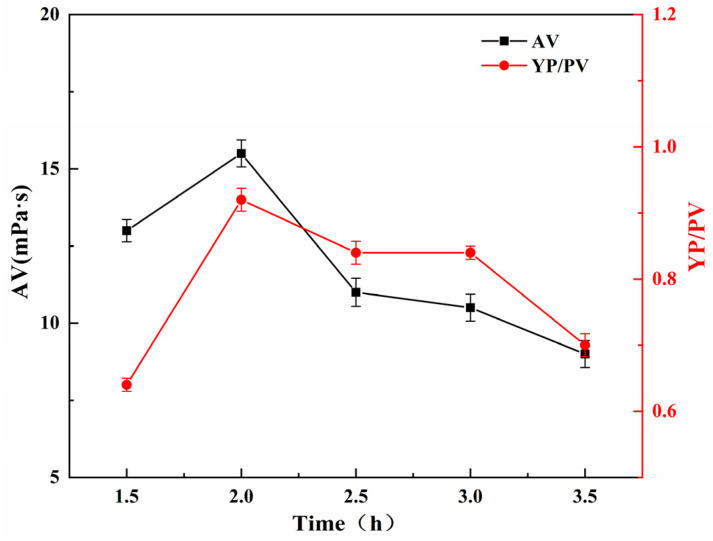
Effect of reaction time on the YP/PV and AV of the SGG.

The effect of reaction time on the YP/PV and AV of the SGG was investigated while maintaining constant reaction conditions: temperature at 26 °C, NaOH dosage at 1.0 wt%, and sulfonating agent dosage at 0.5 wt% (relative to GG mass). As shown in Figure 5, when the reaction time increased from 1.5 h to 2.0 h, both the YP/PV and AV increased markedly, reaching maximum values at 2.0 h. Further extending the reaction time led to a gradual decline in both parameters. This trend indicates that the water-insoluble content was minimized and the modification efficiency was optimized at a reaction time of 2.0 h. This behavior can be attributed to the competing processes during the reaction. Initially, prolonged reaction time promotes the grafting of sulfonate groups onto the GG backbone. These strongly hydrophilic groups enhance electrostatic repulsion and hydration, leading to expanded polymer chains, reduced intermolecular association, lower insoluble content, and improved rheological properties. However, beyond the optimum duration, alkaline-induced hydrolysis of the glycosidic bonds likely becomes dominant, resulting in molecular chain scission, decreased molecular weight, and consequent loss of viscosity. Therefore, the optimal reaction time is determined to be 2.0 h.

#### 2.5.2. Effect of Reaction Temperature on the Performance of SGG

**Figure 6:**
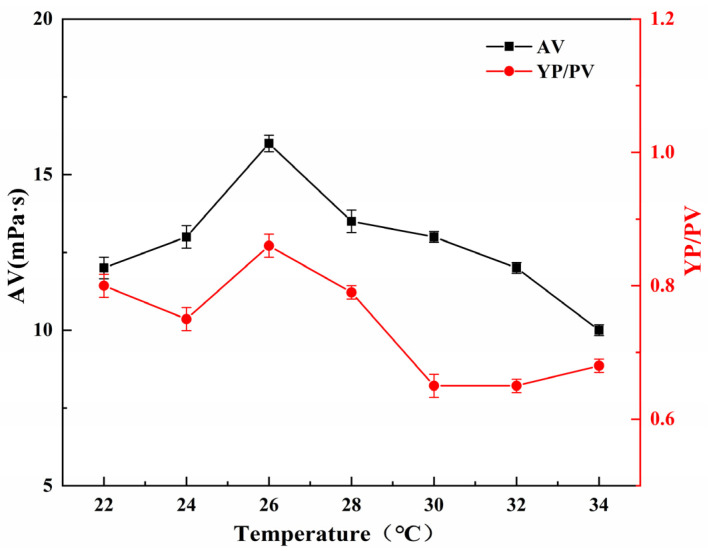
Effect of temperature on the YP/PV and AV of the SGG.

The influence of reaction temperature on the YP/PV and AV of SGG was examined under fixed conditions: reaction time of 2.0 h, NaOH dosage of 1.0 wt%, and sulfonating agent dosage of 0.5 wt% (relative to GG mass). As illustrated in Figure 6, both the YP/PV and AV initially increased with rising temperature from 22 °C to 26 °C, reaching their maximum values at 26 °C. Further increasing the temperature to 34 °C resulted in a gradual decrease in both parameters. The observed behavior can be explained by the dual effects of temperature on the reaction. On one hand, elevated temperature enhances molecular motion and chain expansion, which improves the accessibility of active hydroxyl groups to reactants and thus promotes the sulfonation reaction. On the other hand, beyond a certain threshold, excessive thermal energy may induce colloidal phase separation or aggregation, creating steric hindrance that impedes effective mass transfer and reactant diffusion. This trend indicates that the modification efficiency was optimized at 26 °C.

#### 2.5.3. Effect of Sulfonating Agent Dosage on the Performance of SGG

**Figure 7:**
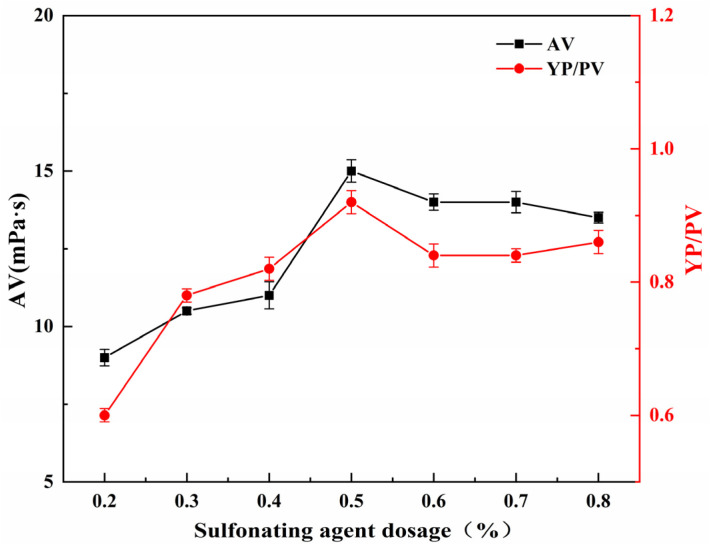
Effect of sulfonating agent dosage on the YP/PV and AV of the SGG.

The effect of sulfonating agent dosage on the YP/PV and AV of SGG was studied under fixed conditions: reaction time of 2.0 h, temperature of 26 °C, and NaOH dosage of 1.0 wt% (relative to GG mass). As shown in Figure 7, both the YP/PV and AV increased with the sulfonating agent dosage from 0.2 wt% to 0.5 wt%, reaching maximum values at 0.5 wt%. Further increasing the dosage beyond 0.5 wt% led to a slight decline in both parameters. The initial increase in rheological properties can be attributed to the progressive grafting of sulfonate groups onto the guar gum backbone, which enhances hydration and chain expansion. However, at higher dosages, the introduced sulfonate groups may induce steric hindrance, limiting the accessibility of remaining reactive hydroxyl groups to the sulfonating agent. The combination of these effects likely explains the observed decrease in AV and YP/PV beyond the optimum dosage. Therefore, the optimal sulfonating agent dosage is determined to be 0.5 wt%.

#### 2.5.4. Effect of NaOH Dosage on the Performance of SGG

**Figure 8:**
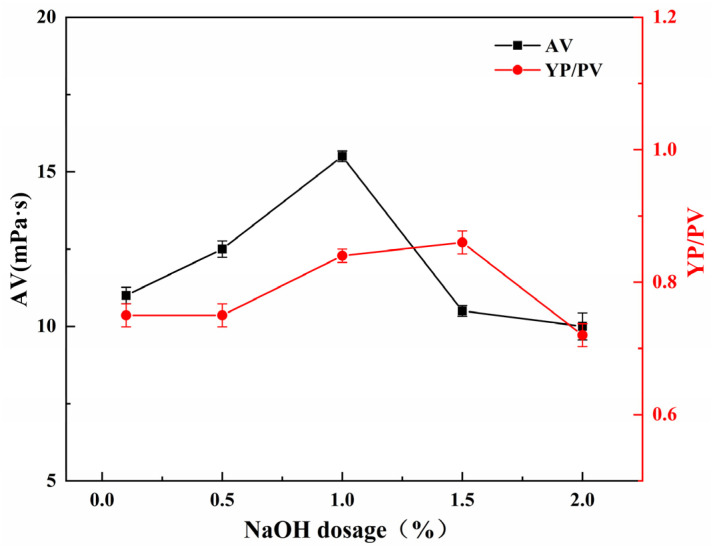
Effect of NaOH dosage on the YP/PV and AV of the SGG.

The influence of NaOH dosage on the YP/PV ratio and AV of SGG was investigated under fixed reaction conditions: 2.0 h, 26 °C, and a sulfonating agent dosage of 0.5 wt% (relative to GG mass). Experiments were conducted with NaOH loadings ranging from 0.1 wt% to 2.0 wt%. As shown in Figure 8, both the YP/PV and AV increased as the NaOH dosage was raised from 0.1 wt% to 1.0 wt%, with AV reaching a maximum at 1.0 wt%. Further increasing the NaOH dosage to 2.0 wt% resulted in a sharp decrease in AV. Although the highest YP/PV value was recorded at 1.5 wt% NaOH, this was primarily a consequence of the drastic reduction in PV, as the actual YP at this loading was lower than that achieved at 1.0 wt% NaOH. This optimal value is attributed to a balance between reaction promotion and polymer degradation. An increase in NaOH concentration enhances catalytic efficiency of the reaction, lowering the reaction activation energy and favoring the formation of the target SGG product. However, when the NaOH concentration exceeds a critical threshold, the strongly alkaline environment induces uncontrolled hydrolysis of the glycosidic bonds in the guar gum backbone. This side reaction leads to scission of the polymer chains, a reduction in molecular weight, and a consequent reduction in viscosity. Therefore, the optimal NaOH dosage is determined to be 1.0 wt%.

### 2.6. Drilling Fluid Performance of SGG

The rheological properties of GG and SGG solutions at room temperature were evaluated to assess the sulfonation modification. Aqueous solutions with concentrations ranging from 0.1 wt% to 0.5 wt% were prepared, and their rheological parameters, including AV, PV, YP, YP/PV, *n*, and K were measured. Comparative analysis of the data in Table 2 indicates that SGG solutions consistently outperformed their GG counterparts at equivalent concentrations. Specifically, the SGG solutions exhibit a significant increase in the YP/PV and YP, while the PV remains largely unchanged. This rheological profile demonstrates the desirable “low-viscosity, high-shear” characteristic of the sulfonated product. For instance, at a concentration of 0.3 wt%, the YP/PV of SGG reached 0.86, representing an increase of 34% compared to the value of 0.64 for native GG. Furthermore, analysis of the n revealed enhanced shear-thinning behavior for SGG. At the 0.3 wt% concentration, the n-value for SGG was 0.45, lower than the value of 0.52 for GG. A lower n-value indicates a greater deviation from Newtonian flow and more pronounced shear-thinning, which is a favorable property for drilling fluids as it improves hydraulic efficiency during circulation.

Following the evaluation of pure gel solutions, the rheological behavior at room temperature of GG and SGG was further investigated in a 4 wt% calcium bentonite base mud. The base mud was aged prior to the addition of gum samples. Comparative analysis of the data in Table 3 demonstrates that the incorporation of gum into the base mud significantly enhanced its rheological properties. Owing to the increased solid content, both the PV and AV rose with increasing gum concentration. More importantly, the YP/PV also showed a substantial increase, rising from 0.42 to 0.93 for GG and from 0.42 to 1.25 for SGG as the concentration increased from 0.1 wt% to 0.5 wt%. At any given concentration, the SGG-based drilling fluid exhibited a markedly higher YP and YP/PV than its GG-based counterpart, while the viscosity remained comparable. This performance profile suggests that the sulfonation modification facilitates the formation of a reinforced gel network structure within the drilling fluid, enhancing intermolecular interactions and thereby improving the fluid’s ability to suspend cuttings.

The fluid loss (FL) and lubrication properties were also evaluated. The FL volume decreased with increasing gum concentration, reaching 15.5 mL for GG and 12.6 mL for SGG at 0.5 wt% concentration, indicating superior fluid loss control for the modified product. In a 4 wt% Ca-bentonite mud, the SGG gel film exhibited a 12% reduction in API fluid loss at a concentration of 0.3 wt%. A qualitative analysis of the filter cakes revealed that the SGG-based fluid formed a thinner, tougher, and more compact filter cake compared to the thicker, more porous cake formed by the GG-based fluid, consistent with its lower fluid loss values. The friction coefficient values were similar for both fluids at optimal concentrations (e.g., 0.09 at 0.3 wt% addition), suggesting satisfactory lubricity. The gel strength values (G10s and G10min) at 0.3 wt% concentration (1.0 Pa and 1.5 Pa, respectively) indicate good thixotropy, ensuring adequate suspension capabilities at low shear rates in the annulus without generating excessively high pump pressure upon resumption of circulation. Considering the overall performance, excessively high viscosity may impede bit cleaning, while insufficient viscosity compromises cuttings transport. Therefore, a 0.3 wt% gum concentration is considered optimal for the 4 wt% calcium bentonite base mud, with SGG providing superior rheological enhancement and fluid loss control.

**Figure 9:**
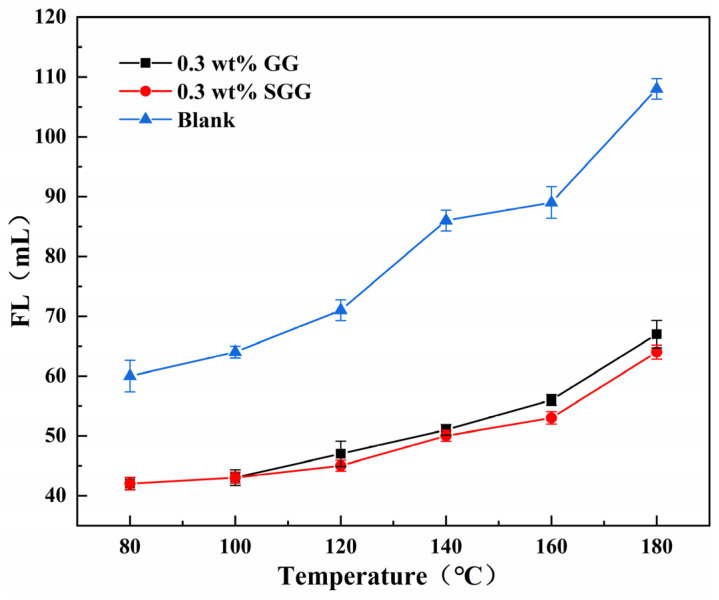
Fluid loss of GG-based and SGG-based drilling fluid at various temperatures.

**Figure 10:**
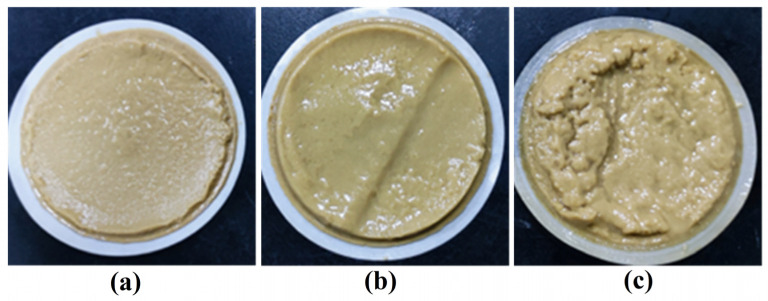
Filter cakes of SGG-based drilling fluid at (**a**) 80 °C, (**b**) 120 °C, and (**c**) 180 °C.

The high pressure and temperature fluid loss behavior of the drilling fluids was further investigated. Formulations containing different concentrations of GG or SGG in a 4 wt% calcium bentonite base mud were tested under a pressure of 3.5 MPa across a temperature range of 80 to 180 °C. As shown in Figure 9, the fluid loss was significantly reduced for both GG-based and SGG-based fluids compared to the pure base mud. The fluid loss volume increased gradually at temperatures below 140 °C, with a minor difference observed between the modified and unmodified samples. However, beyond 140 °C, a more pronounced increase in fluid loss was observed. Notably, the SGG-based fluids consistently exhibited lower fluid loss than the GG-based fluids across the entire temperature range, demonstrating the enhanced temperature resistance imparted by sulfonation. The filter cakes formed at 180 °C (Figure 10) appeared loose and poorly structured, indicating severe degradation of the fluid composition at extreme temperatures, which corresponds to the sharp rise in fluid loss.

### 2.7. Evaluation of Temperature Resistance

**Figure 11:**
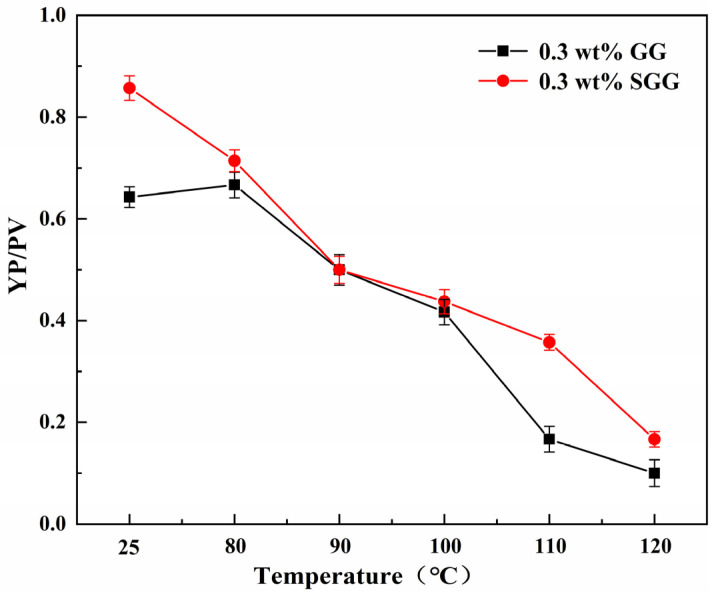
Effect of temperature on the YP/PV ratio of gel solutions.

**Figure 12:**
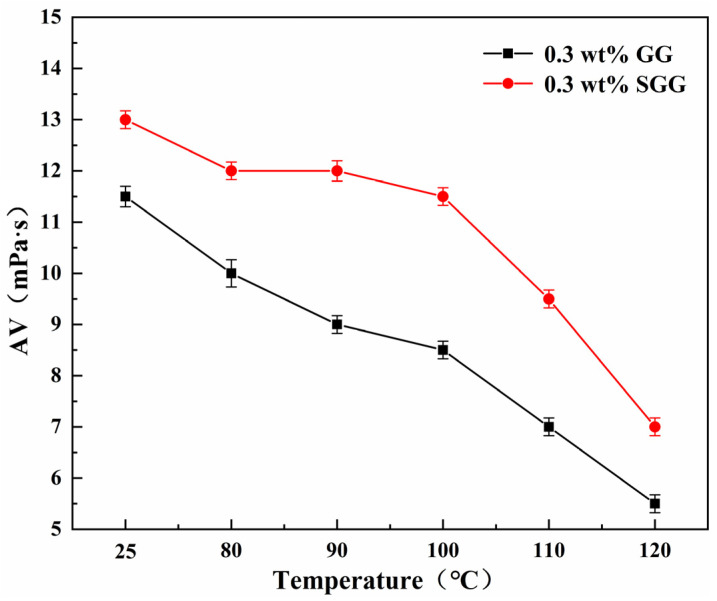
Effect of temperature on the AV of gel solutions.

The thermal stability of GG and SGG was assessed by hot-rolling the samples for 16 h at temperatures ranging from 80 °C to 120 °C. The rheological properties of the gel solutions after thermal aging are summarized in Table 4 and illustrated in Figure 11 and Figure 12. The results indicate a progressive deterioration in the rheological properties of both gels with increasing aging temperature, marked by a significant decline in YP, AV, and YP/PV. However, at any given temperature, the SGG solutions consistently retained higher rheological performance compared to the GG, confirming that sulfonation effectively enhances the thermal stability of guar gum, allowing it to maintain better functionality under elevated temperatures. A notable inflection point was observed between 90 °C and 100 °C, where the rate of degradation slowed. This suggests that the disruption of the gel’s network structure within this temperature range may be partially reversible, allowing for some recovery of properties over time. In contrast, beyond 110 °C, the properties declined sharply, indicating irreversible degradation of the gel structure.

**Figure 13:**
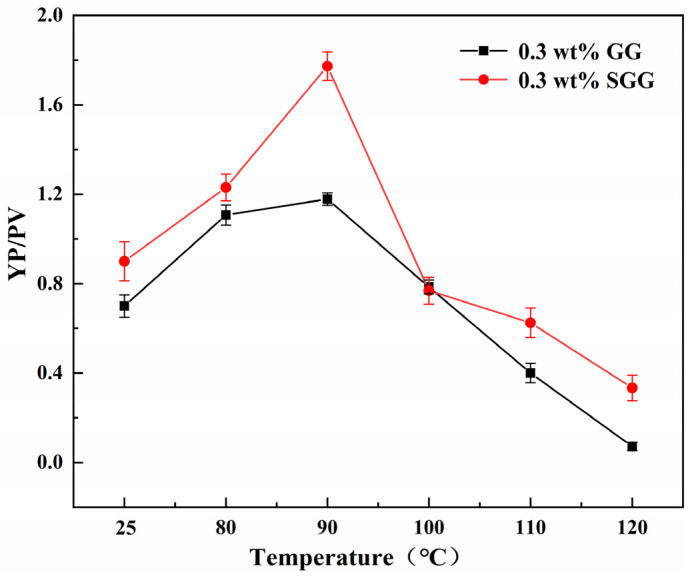
Effect of temperature on the YP/PV ratio of GG-based and SGG-based drilling fluids.

**Figure 14:**
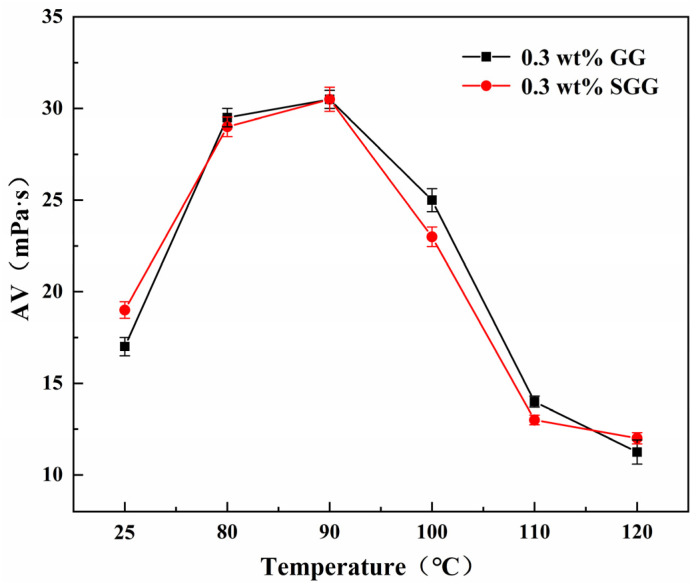
Effect of temperature on the AV of GG-based and SGG-based drilling fluids.

To better evaluate the performance of modified guar gum under practical conditions, the temperature resistance of GG and SGG was further investigated in a 4 wt% calcium bentonite base mud. The base mud was aged prior to use. Drilling fluid formulations containing GG or SGG were subjected to hot-rolling aging for 16 h at temperatures ranging from 80 °C to 120 °C. The results are presented in Table 5, Figure 13 and Figure 14. The rheological properties of both the GG and SGG drilling fluids exhibited a non-monotonic response to increasing aging temperature, characterized by an initial increase followed by a subsequent decrease. The YP/PV, AV and gel strength increased with temperature up to 90 °C, reaching a maximum at this point. The SGG-based fluid achieved a notably high YP/PV of 1.77 at 90 °C, demonstrating excellent shear-thinning behavior, whereas the GG-based fluid reached only 1.18 under the same conditions. Beyond 90 °C, all properties declined with further increases in temperature. Importantly, the SGG fluid exhibited a higher retention of the YP/PV after the peak compared to the GG fluid. At 120 °C, the YP/PV of the SGG fluid was 4.7 times that of the GG fluid, clearly indicating the thermal stability of conferred by sulfonation. The notably largest difference in YP/PV between SGG and GG observed at 90 °C (Figure 13) can be understood as a competition between thermal activation and thermal degradation. At this temperature, the SGG network appears to be thermally activated, with enhanced molecular motion promoting chain unfolding and optimal network development. In contrast, the native GG network, lacking the stabilizing sulfonate groups, begins to undergo irreversible thermal degradation through hydrolysis of its glycosidic bonds.

The initial improvement in rheological properties with temperature can be attributed to enhanced molecular motion, which promotes the uncoiling of the polymer chains and reduces the content of water-insoluble components. This leads to an increase in hydrodynamic volume and consequently enhances shear-thinning behavior. The introduced sulfonate groups further strengthen the hydration layer around the polymer chains and help stabilize the resulting gel network structure, thereby delaying thermal degradation. This mechanism allows the SGG fluid to maintain a higher YP/PV over a broader temperature range. In conclusion, sulfonation modifies the guar gum structure, improving its hydration stability and overall performance at elevated temperatures, thereby significantly enhancing its suitability for high-temperature drilling applications.

### 2.8. Evaluation of Salt Tolerance

**Figure 15:**
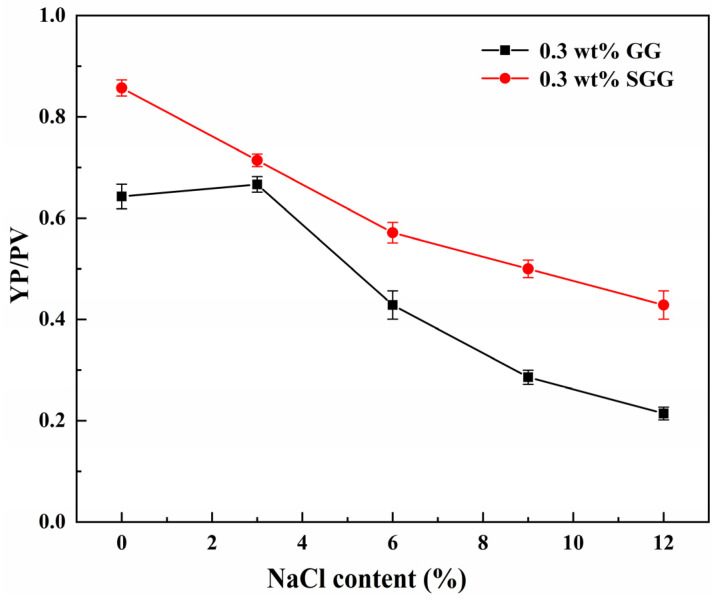
Effect of NaCl content on the YP/PV ratio of the gel solution.

To evaluate the salt tolerance of guar gum before and after modification, the rheological properties of both GG and SGG were tested in pure gel solutions with varying NaCl concentrations at 25 °C. The results are presented in Table 6 and Figure 15. The introduction of NaCl significantly reduced the AV, YP, and YP/PV in both fluids. However, SGG demonstrated markedly superior salt tolerance, maintaining better gel integrity. At equivalent salt concentrations, the YP/PV and YP of SGG were approximately 30% to 60% higher than those of GG. The YP/PV of GG exhibited a nonlinear response to increasing NaCl content, peaking at 3 wt% NaCl. This peak arises because the YP/PV reflects the structural strength and shear-thinning efficiency at a given viscosity. The cations from NaCl shield the electrostatic repulsion between GG molecular chains, thereby suppressing hydration. In contrast, the strongly electronegative sulfonate groups introduced by sulfonation enhance interchain electrostatic repulsion, mitigating the cationic shielding effect. This helps preserve the hydration layer and maintain gel network stability around the SGG chains even in high-salinity environments, allowing them to maintain stronger shear-thinning characteristics and structural integrity.

**Figure 16:**
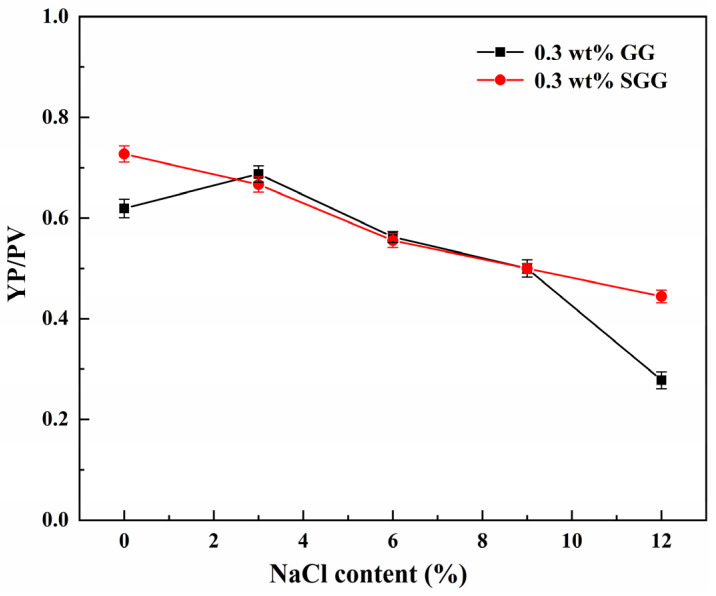
Effect of NaCl content on the YP/PV ratio of GG-based and SGG-based drilling fluids.

The performance of GG and SGG was further assessed in actual drilling fluids. The presence of clay particles and other additives introduces potential interactions that may alter salt tolerance. Therefore, the salt tolerance of GG and SGG was evaluated in a 4 wt% calcium bentonite base mud, with results shown in Table 7 and Figure 16. Compared to the pure gel solutions, the drilling fluid formulations exhibited significantly higher viscosity due to the increased solid content. The Ca^2+^ from the calcium bentonite likely formed ionic bridges with hydroxyl or carboxyl groups on the guar gum chains, reinforcing a three-dimensional gel network. Additionally, the bentonite particles adsorbed gel polymer chains, forming a “polymer-bentonite” composite structure. The introduction of NaCl markedly reduced the AV, YP, and YP/PV in both the GG and SGG drilling fluids. However, at each NaCl concentration, the SGG-based fluid maintained superior rheological properties compared to the GG-based fluid. The YP/PV ratio of the SGG remained at 0.44 even at a NaCl concentration of 12 wt%, representing a 57% enhancement over the native GG. This demonstrates that the enhanced salt tolerance of the gel structure achieved through sulfonation remains effective in the complex drilling fluid environment, indicating consistent performance unaffected by other fluid components. Notably, the YP/PV of the GG drilling fluid showed minimal sensitivity to NaCl concentrations up to 9 wt%, but decreased sharply at 12 wt%, suggesting that the integrity of the gel network had been compromised. In contrast, the SGG drilling fluid exhibited only a gradual decline in YP/PV even at 12 wt% NaCl, confirming its exceptional salt tolerance in practical drilling fluid applications. The observed more pronounced drop in gel strength for SGG at high salinity (Table 7) is attributed to the time-dependent relaxation of its electrostatically reinforced network upon charge shielding by ions, which is a characteristic of this type of anionic polymer. Nevertheless, the consistently higher YP/PV ratio of SGG under all saline conditions demonstrates that its overall suspension capacity and shear-thinning behavior remain superior to native GG.

### 2.9. Evaluation of Inhibition Performance

The inhibition capability of drilling fluids is crucial for maintaining wellbore stability and ensuring safe and efficient drilling operations. When penetrating shale and other water-sensitive formations, the invasion of drilling fluid filtrate can induce hydration, swelling, and dispersion of formation rocks, leading to wellbore instability, narrowing, or even collapse.

**Figure 17:**
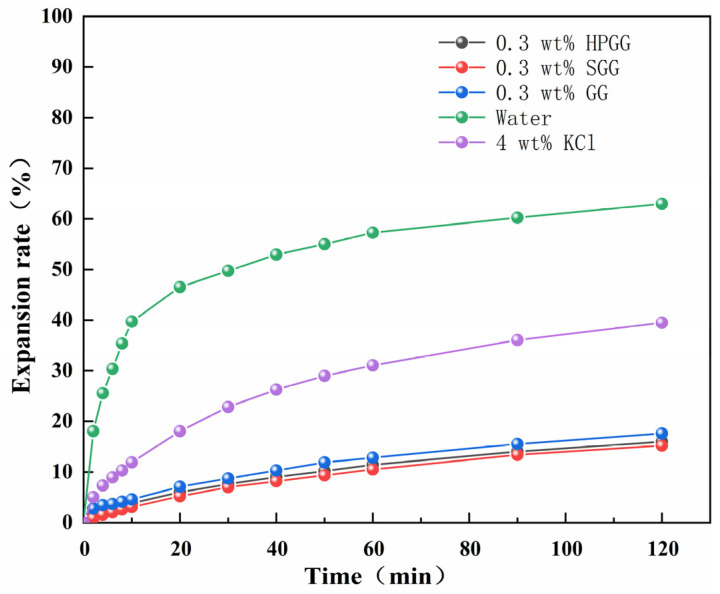
Linear expansion test results of GG and SGG.

The inhibition performance of the prepared SGG was evaluated using a linear expansion test at 25 °C, with results shown in Figure 17. The bentonite swelling rates after 120 min of hydration in deionized water and a 4 wt% KCl solution were 62.98% and 39.12%, respectively, serving as reference values. In contrast, both the native GG and SGG solutions exhibited significantly lower swelling rates, with the SGG sample demonstrating the best inhibition performance. SGG demonstrated a 14% improvement in inhibition, with a final expansion rate of 15.21% after 120 min, significantly lower than GG’s 17.61%. The superior inhibition of both fluids compared to water and KCl can be attributed to two primary mechanisms. First, the hydroxyl groups on the guar gum chains form hydrogen bonds with water molecules, which inhibits and retards water penetration into the clay interlayers. Second, the gel polymer chains adsorb onto the surface of clay particles, forming a dense coating that hinders their hydration and dispersion. The further enhancement in inhibition observed for SGG is due to the introduction of highly electronegative –SO_3_H. These groups enhance hydration by strengthening the electrostatic repulsion between molecular chains and increase the rigidity of the adsorbed polymer layer, with a consequent improvement in its stability on the clay surface and inhibition capability.

### 2.10. Mechanism Discussion

**Figure 18:**
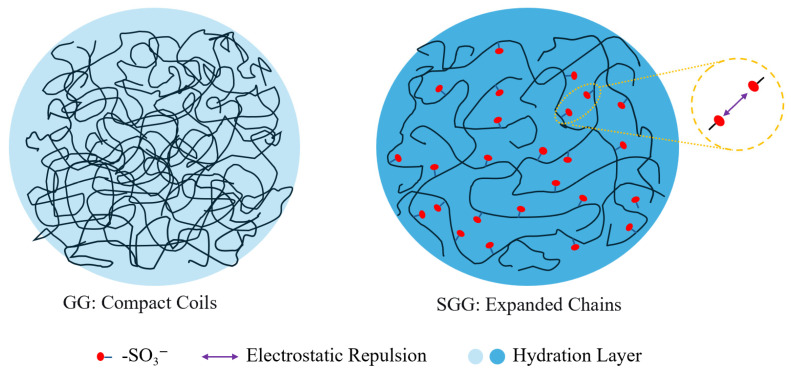
Schematic illustration of the molecular structure evolution from GG to SGG.

The proposed mechanism for the enhanced performance of SGG is schematically illustrated in Figure 18. In its native state, GG molecules adopt compact coil conformations in aqueous solution, which results in limited chain expansion, weak electrostatic repulsion, and a relatively thin hydration layer, which collectively restricts its rheological stability, particularly under demanding conditions of high temperature and salinity. The chemical introduction of -SO_3_^−^ via sulfonation fundamentally alters this molecular architecture. The strongly anionic and hydrophilic sulfonate moieties introduce two critical repulsive forces: significant electrostatic repulsion due to their negative charges, and substantial steric hindrance due to their molecular volume. The combination forces the polymer chains to undergo a dramatic expansion from their initial compact coils into more extended conformations. This molecular-level expansion is directly reflected in the macroscopic rheological data. The expanded chains occupy a larger hydrodynamic volume and form a more robust network, which is quantified by the 34% increase in the YP/PV ratio (from 0.64 to 0.86 for a 0.3 wt% gel, Table 2) and the decrease in the flow behavior index (n) from 0.52 to 0.45 (Table 2). A lower n-value is a direct quantitative indicator of more pronounced shear-thinning, which is a classic signature of a fluid containing expanded, interacting polymer chains.

The expanded chains occupy a larger hydrodynamic volume, which directly contributes to a more robust and resilient three-dimensional gel network. Crucially, the grafted sulfonate groups foster the formation of a thick, persistent hydration layer via their powerful hydrophilic interactions with water molecules. This enhanced hydration, coupled with the continuous electrostatic repulsion that helps maintain network spacing, grants the SGG gel exceptional stability against the dehydrating and screening effects of high temperatures and elevated salt concentrations. The performance of SGG under harsh conditions provides quantitative support for this enhanced hydration. Superior hydration stability resists thermal dehydration, as shown by the 4.7-fold higher retention of YP/PV after aging at 120 °C (0.33 for SGG vs. 0.07 for GG, Table 5). Similarly, the sustained electrostatic repulsion helps counter ionic screening, evidenced by the 57% higher YP/PV maintained in 12 wt% NaCl brine (0.44 for SGG vs. 0.28 for GG, Table 7).

Consequently, this mechanism accounts for the observed “low-viscosity, high-shear” rheological profile, improved fluid loss control, and outstanding retention of key properties in harsh environments, as demonstrated experimentally.

## 3. Conclusions

This study successfully synthesized SGG through alkaline etherification using sodium 3-chloro-2-hydroxy-propane sulfonate. The successful introduction of sulfonate groups onto the GG backbone was unequivocally confirmed by FTIR, EA and SEM. TGA further demonstrated enhanced thermal stability of the polymer. The sulfonation process was optimized, with the ideal reaction conditions identified as 26 °C for 2.0 h using 1.0 wt% NaOH and 0.5 wt% sulfonating agent.

Comprehensive performance evaluations confirmed that SGG functions as a highly effective rheology modifier for water-based drilling fluids. It delivers a desirable low-viscosity, high-shear profile, elevating the YP/PV ratio by 34% and markedly enhancing shear-thinning behavior and suspension capacity without substantially increasing PV. In a 4 wt% calcium bentonite mud, SGG reduced API fluid loss by 12% and maintained a YP/PV of 0.33 after 120 °C aging, representing a 4.7-fold retention over GG. Furthermore, SGG sustained a YP/PV of 0.44 in 12 wt% NaCl brine and improved inhibition by 14%. These enhancements are attributed to the strong hydration capacity and electrostatic repulsion introduced by the sulfonate groups, which foster a more robust and stable three-dimensional gel network within the fluid. Therefore, SGG presents a promising gel additive for formulating high-performance drilling fluids, particularly for applications in high-temperature and high-salinity environments. While this work has conclusively demonstrated its efficacy up to 120 °C, evaluating the performance of SGG at temperatures exceeding this range will be a key focus of future work. Such a study could be extended to include a comparative performance evaluation against other commercial and modified bio-polymers to further benchmark its advantages.

## 4. Materials and Methods

### 4.1. Synthesis of Sodium 3-Chloro-2-Hydroxypropanesulfonate

**Figure 19:**
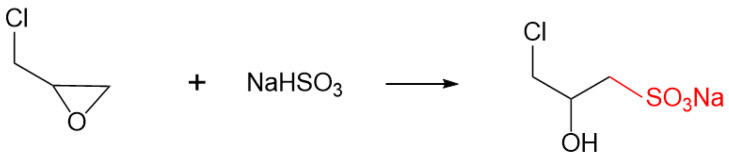
Reaction scheme for the synthesis of 3-chloro-2-hydroxypropanesulfonate.

Sodium bisulfite (analytical grade) was purchased from Jinan Kunfeng Chemical Co., Ltd. (Jinan, China). Epichlorohydrin (analytical grade) was obtained from Shandong Xinrongxin Chemical Technology Co., Ltd. (Jinan, China). The sulfonating agent, sodium 3-chloro-2-hydroxypropanesulfonate, was synthesized as follows: A solution of sodium bisulfite in water was prepared in a three-necked flask under magnetic stirring. Epichlorohydrin was then added to the solution, and the reaction was carried out at a set temperature with continuous stirring. Upon completion, the final product was isolated by removing the residual water and unreacted sodium bisulfite using a rotary evaporator. The chemical reaction scheme is shown in Figure 19.

### 4.2. Sulfonation of GG

**Figure 20:**
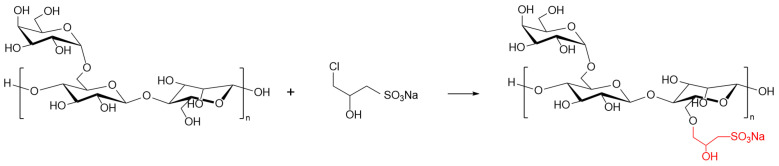
Reaction scheme for the sulfonation process.

Guar gum powder (technical grade) was supplied by Shandong Chengshun Chemical Technology Co., Ltd. (Jinan, China). Acetic acid (analytical grade) was purchased from Xi’an Chemical Reagent Factory (Xi’an, China). Sodium hydroxide (analytical grade) was obtained from Zhengzhou Pini Chemical Reagent Factory (Zhengzhou, China). Anhydrous ethanol (analytical grade) was sourced from Tianjin Hongyan Reagent Factory (Tianjin, China). The sulfonation of GG was performed as follows: GG powder was dissolved in a mixture of anhydrous ethanol and distilled water in a three-necked flask under stirring. Under a continuous nitrogen atmosphere, sodium hydroxide and the sulfonating agent were added to the solution. The reaction was carried out with mechanical stirring at 300 r/min under reflux in a constant-temperature water bath. Upon completion, the mixture was neutralized to pH = 7 with acetic acid. The product was isolated by suction filtration, washed thoroughly with anhydrous ethanol, and dried at 60 °C to constant weight. The resulting solid was ground into a powder to yield SGG. The chemical reaction scheme is shown in Figure 20. The performance evaluation for each synthesis condition was conducted in triplicate.

### 4.3. FTIR Measurement

Fourier transform infrared (FTIR) spectroscopy analysis of the SGG sample was performed on a Thermo Nicolet 5700 spectrometer (Madison, WI, USA). The solid sample was prepared by mixing 1–2 mg of the dried product with 150 mg of spectroscopic-grade KBr, followed by grinding into a homogeneous fine powder and compressing into a transparent pellet under a pressure of 10 MPa. Spectra were acquired at a resolution of 4 cm^−1^ with 32 scans over the wavenumber range of 4000–400 cm^−1^.

### 4.4. EA Measurement

Elemental analysis (EA) of the GG powder was conducted using a Vario MICRO Cube elemental analyzer (Frankfurt, Germany). Prior to analysis, the sample was ground into a fine powder and dried in a vacuum oven at 60 °C for 24 h to remove moisture and volatile impurities. For measurement, 2–3 mg of the prepared sample was accurately weighed and compacted into a sample boat. The analysis was performed under the following conditions: combustion tube temperature at 1150 °C, reduction tube at 850 °C, and a carrier gas flow rate of 200 mL/min, with oxygen injection dynamically adjusted based on sample carbon content. The sample was combusted in an oxygen atmosphere, and the resulting gases were separated by chromatography and quantified using a thermal conductivity detector.

### 4.5. TGA Measurement

Thermogravimetric analysis (TGA) was performed using a TGA/DSC1 analyzer from Mettler Toledo (Greifensee, Switzerland). A sample mass of 5–10 mg was placed in an alumina crucible and heated from ambient temperature to 800 °C at a rate of 15 °C/min under a nitrogen atmosphere with a flow rate of 20 mL/min.

### 4.6. SEM Measurement

Scanning electron microscopy (SEM) was performed on a Hitachi SU8600 FE-SEM instrument (Tokyo, Japan). Samples were platinum-coated to ensure conductivity and imaged using a secondary electron detector at 3 kV and a working distance of 12 mm.

### 4.7. Rheological Properties Measurement

The rheological properties of the drilling fluids, including plastic viscosity (PV), yield point (YP), apparent viscosity (AV), and gel strengths, were determined using a ZNN-D12 digital rotary viscometer (Qingdao Haitongda Specialized Instrument Factory, Qingdao, China) [33]. The sample was filled to the marked line in the viscometer cup. The dial readings at 600 and 300 r/min (Φ_600_ and Φ_300_) were recorded after values stabilized. The gel strengths at 10 s (G_10s_) and 10 min (G_10min_) were measured as the maximum dial reading at 3 r/min after shearing the fluid at 600 r/min for 10 s, followed by respective rest periods of 10 s and 10 min. The PV, YP, and AV were calculated using the following Equations (1)–(4).

(1) $$PV=\Phi_{600}-\Phi_{300}$$

(2) $$YP=\frac{1}{2}\left(\Phi_{300}-PV\right)$$

(3) $$AV=\frac{1}{2}\Phi_{600}$$

(4) $$G_{10s}\;or\;G_{10min}=\frac{1}{2}\Phi_{3}$$

PV: plastic viscosity, mPa·s;

AV: apparent viscosity, mPa·s;

YP: yield point, Pa;

G_10s_ or G_10min_: gel strength at 10 s or 10 min, Pa;

Φ_600_, Φ_300_: steady dial reading at 600 r/min or 300 r/min;

Φ_3_: maximum dial reading at 3 r/min after a rest period of 10 s or 10 min.

The drilling fluid’s rheology was modeled using the power-law model to describe its annular flow behavior. The flow behavior index (n) is a dimensionless measure of the fluid’s deviation from Newtonian behavior. The consistency index (K) serves as a direct indicator of the fluid’s thickness or effective viscosity. As shown in Equations (5) and (6).

(5) $$n=3.322lg\left(\frac{\Phi_{600}}{\Phi_{300}}\right)$$

(6) $$K=0.5\frac{\Phi_{300}}{500^{n}}$$

### 4.8. Fluid Loss Properties Measurement

The fluid loss properties of the GG drilling fluid were evaluated under both ambient and high-pressure/high-temperature conditions. The API fluid loss at ambient temperature was measured using an SD-type multi-unit filter (Qingdao Haitongda Specialized Instrument Factory, Qingdao, China) press pressurized to 0.69 MPa. The filtrate volume collected over 7.5 min was recorded as the API fluid loss, and the resulting filter cake was retained for subsequent lubricity analysis. The HPHT fluid loss was determined using a GGS42-Z HPHT filter (Qingdao Haitongda Specialized Instrument Factory, Qingdao, China) press at 3.5 MPa and elevated temperature. A backpressure of 100 psi (690 kPa) was maintained throughout the test via the filter liquid receiver to prevent fluid boiling. The filtrate volume was collected over 30 min.

### 4.9. Lubricity Measurement

The lubricity of the drilling fluid was evaluated by measuring the coefficient of friction of the filter cake. The test was conducted using an NZ-3A lubricity tester (Qingdao Haitongda Specialized Instrument Factory, Qingdao, China) on the filter cake obtained from the preceding API fluid loss test.

### 4.10. Thermal Stability Measurement

The drilling fluid samples were hot rolled in aging cells filled to the calibration line. The cells were placed in a rolling oven and aged at temperatures of 80, 90, 110, and 120 °C for 16 h. Subsequently, the samples were cooled to room temperature prior to further performance evaluation.

### 4.11. Salt Tolerance Measurement

NaCl and KCl were supplied by Tianjin Shengao Chemical Reagent Co., Ltd. (Tianjin, China). Bentonite (technical grade) was obtained from Xi’an Permanent Chemical Co., Ltd. (Xi’an, China). Saltwater base muds were prepared by adding various mass fractions (3 wt%, 6 wt%, 9 wt%, and 12 wt%) of KCl or NaCl to 350 mL of freshwater base mud under high-speed stirring. The mixtures were then hydrated for 4 h. Following the addition of the target additives, the muds were aged for 16 h, after which their salt tolerance was evaluated by measuring the resultant rheological properties.

### 4.12. Inhibition Properties Measurement

The inhibition properties were evaluated using an NP-01 linear expansion meter (Qingdao Hengtaida Mechanical & Electrical Equipment Co., Ltd., Qingdao, China) [34,35]. A pellet was first prepared by compressing 10 g of oven-dried sodium bentonite under 10 MPa for 10 min. The pellet was then placed in the tester, the dial gauge was zeroed, and the test solution was poured in. The swelling amount was recorded at designated time points, and the swelling percentage was calculated relative to the initial pellet thickness.

## Figures and Tables

**Table 1:** Elemental analysis results.

			Content/%		
	C	N	H	S	Other
GG	39.800	0.526	5.998	0.621	53.055
SGG	38.500	0.515	5.345	0.792	54.848

**Table 2:** Rheological properties of GG and SGG gel solutions. Values are the mean + standard deviation of three replicates.

	AV/mPa·s	PV/mPa·s	YP/Pa	YP/PV	*n*	K/Pa·sn
0.1 wt% GG	4.50	3.00	1.50	0.50	0.58	0.08
0.2 wt% GG	9.50	6.00	3.50	0.58	0.55	0.22
0.3 wt% GG	11.50	7.00	4.50	0.64	0.52	0.31
0.4 wt% GG	17.00	10.00	7.00	0.70	0.50	0.53
0.5 wt% GG	24.00	12.00	12.00	1.00	0.42	1.36
0.1 wt% SGG	4.50	3.00	1.50	0.50	0.58	0.08
0.2 wt% SGG	10.00	6.00	4.00	0.67	0.51	0.29
0.3 wt% SGG	13.00	7.00	6.00	0.86	0.45	0.57
0.4 wt% SGG	17.50	9.00	8.50	0.94	0.43	0.90
0.5 wt% SGG	24.00	11.00	13.00	1.18	0.38	1.79

**Table 3:** Rheological properties of 4 wt% calcium bentonite base mud treated with varying concentrations of GG and SGG. Values are the mean + standard deviation of three replicates.

	AV/mPa·s	PV/mPa·s	YP/Pa	YP/PV	G_10s_/Pa	G_10min_/Pa	FL/mL	Friction Coefficient
0.1 wt% GG	8.50	6.00	2.50	0.42	0.50	1.50	17.80	0.16
0.2 wt% GG	12.00	8.00	4.00	0.50	0.50	1.50	16.50	0.14
0.3 wt% GG	17.00	10.50	6.50	0.62	1.00	1.50	16.30	0.09
0.4 wt% GG	19.00	10.50	8.50	0.81	1.50	2.00	16.00	0.11
0.5 wt% GG	27.00	14.00	13.00	0.93	2.50	3.00	15.50	0.13
0.1 wt% SGG	8.50	6.00	2.50	0.42	0.50	1.50	16.60	0.18
0.2 wt% SGG	12.00	7.00	5.00	0.71	0.50	1.50	15.80	0.13
0.3 wt% SGG	19.00	10.00	9.00	0.90	1.00	1.50	14.30	0.09
0.4 wt% SGG	20.00	10.00	10.00	1.00	1.50	2.00	13.50	0.12
0.5 wt% SGG	27.00	12.00	15.00	1.25	2.50	3.00	12.60	0.12

**Table 4:** Effect of temperature on the rheological properties of gel solutions. Values are the mean + standard deviation of three replicates.

Temperature/°C		AV/mPa·s	PV/mPa·s	YP/Pa	YP/PV	G_10s_/Pa	G_10min_/Pa
25	0.3 wt% GG	11.50	7.00	4.50	0.64	0.50	0.50
	0.3 wt% SGG	13.00	7.00	6.00	0.86	0.50	0.50
80	0.3 wt% GG	10.00	6.00	4.00	0.67	0.25	0.25
	0.3 wt% SGG	12.00	7.00	5.00	0.71	0.25	0.25
90	0.3 wt% GG	9.00	6.00	3.00	0.50	0.25	0.25
	0.3 wt% SGG	12.00	8.00	4.00	0.50	0.25	0.25
100	0.3 wt% GG	8.50	6.00	2.50	0.42	0.25	0.25
	0.3 wt% SGG	11.50	8.00	3.50	0.44	0.25	0.25
110	0.3 wt% GG	7.00	6.00	1.00	0.17	0.00	0.00
	0.3 wt% SGG	9.50	7.00	2.50	0.36	0.25	0.25
120	0.3 wt% GG	5.50	5.00	0.50	0.10	0.00	0.00
	0.3 wt% SGG	7.00	6.00	1.00	0.17	0.00	0.00

**Table 5:** Effect of temperature on the rheological properties of GG-based and SGG-based drilling fluids. Values are the mean + standard deviation of three replicates.

Temperature/°C		AV/mPa·s	PV/mPa·s	YP/Pa	YP/PV	G_10s_/Pa	G_10min_/Pa
25	0.3 wt% GG	17.00	10.00	7.00	0.70	1.00	1.50
	0.3 wt% SGG	19.00	10.00	9.00	0.90	1.00	1.50
80	0.3 wt% GG	29.50	14.00	15.50	1.11	4.00	4.50
	0.3 wt% SGG	29.00	13.00	16.00	1.23	4.25	5.25
90	0.3 wt% GG	30.50	14.00	16.50	1.18	4.00	4.50
	0.3 wt% SGG	30.50	11.00	19.50	1.77	4.50	5.50
100	0.3 wt% GG	25.00	14.00	11.00	0.79	2.00	3.00
	0.3 wt% SGG	23.00	13.00	10.00	0.77	2.00	3.00
110	0.3 wt% GG	14.00	10.00	4.00	0.40	0.25	0.50
	0.3 wt% SGG	13.00	8.00	5.00	0.63	0.25	0.50
120	0.3 wt% GG	11.25	10.50	0.75	0.07	0.25	0.25
	0.3 wt% SGG	12.00	9.00	3.00	0.33	0.25	0.25

**Table 6:** Effect of NaCl content on the rheological properties of gel solutions. Values are the mean + standard deviation of three replicates.

	NaCl Content	AV/mPa·s	PV/mPa·s	YP/Pa	YP/PV	G_10s_/Pa	G_10min_/Pa
0.3 wt% GG	0 wt%	11.50	7.00	4.50	0.64	0.50	0.50
	3 wt%	10.00	6.00	4.00	0.67	0.25	0.25
	6 wt%	10.00	7.00	3.00	0.43	0.00	0.00
	9 wt%	9.00	7.00	2.00	0.29	0.00	0.00
	12 wt%	8.50	7.00	1.50	0.21	0.00	0.00
0.3 wt% SGG	0 wt%	13.00	7.00	6.00	0.86	0.50	0.50
	3 wt%	12.00	7.00	5.00	0.71	0.25	0.25
	6 wt%	11.00	7.00	4.00	0.57	0.00	0.00
	9 wt%	10.50	7.00	3.50	0.50	0.00	0.00
	12 wt%	10.00	7.00	3.00	0.43	0.00	0.00

**Table 7:** Effect of NaCl content on the rheological properties of GG-based and SGG-based drilling fluids. Values are the mean + standard deviation of three replicates.

	NaCl Content	AV/mPa·s	PV/mPa·s	YP/Pa	YP/PV	G_10s_/Pa	G_10min_/Pa
0.3 wt% GG	0 wt%	17.00	10.50	6.50	0.62	1.00	1.25
	3 wt%	13.50	8.00	5.50	0.69	1.00	1.25
	6 wt%	12.50	8.00	4.50	0.56	0.50	1.00
	9 wt%	12.00	8.00	4.00	0.50	0.50	1.00
	12 wt%	11.50	9.00	2.50	0.28	0.50	0.75
0.3 wt% SGG	0 wt%	19.00	11.00	8.00	0.73	1.00	1.25
	3 wt%	15.00	9.00	6.00	0.67	1.00	1.00
	6 wt%	14.00	9.00	5.00	0.56	1.00	0.75
	9 wt%	13.50	9.00	4.50	0.50	1.00	0.75
	12 wt%	13.00	9.00	4.00	0.44	0.50	0.50

**NaCl Content**

**AV/mPa·s**

**PV/mPa·s**

**YP/Pa**

**YP/PV**

**G_10s_/Pa**

**G_10min_/Pa**

## Data Availability

The original contributions presented in this study are included in the article. Further inquiries can be directed to the corresponding author.
